# Laser-Milled Microslits Improve the Bonding Strength of Acrylic Resin to Zirconia Ceramics

**DOI:** 10.3390/polym12040817

**Published:** 2020-04-04

**Authors:** Saiji Shimoe, Tzu-Yu Peng, Yuki Wakabayashi, Hiroto Takenaka, Shogo Iwaguro, Masato Kaku

**Affiliations:** 1 Department of Anatomy and Functional Restorations, Integrated Health Sciences, Hiroshima University Graduate School of Biomedical and Health Sciences, Hiroshima 734-8553, Japan; pengtzuyu1014@gmail.com (T.-Y.P.); mkaku@hiroshima-u.ac.jp (M.K.); 2 Department of Dental Medical Laboratory, Hokkaido University Hospital, Sapporo Hokkaido 060-8648, Japan; y.wakabayashi@den.hokudai.ac.jp; 3 Dental Laboratory Center, Department of Medical Technology, Nagasaki University Hospital, Nagasaki 852-8501, Japan; takenaka-h@nagasaki-u.ac.jp; 4 Division of Dental Technician, Department of Clinical Practice and Support, Hiroshima University Hospital, Hiroshima 734-8553, Japan; iwaguro@hiroshima-u.ac.jp

**Keywords:** zirconium oxide, acrylic resin, bond strength, laser, thermocycling

## Abstract

Heightened aesthetic considerations in modern dentistry have generated increased interest in metal-free “zirconia-supported dentures.” The lifespan of the denture is largely determined by the strength of adhesion between zirconia and the acrylic resin. Thus, the effect on shear bond strength (SBS) was investigated by using an acrylic resin on two types of zirconia ceramics with differently sized microslits. Micromechanical reticular retention was created on the zirconia surface as the novel treatment (microslits (MS)), and air-abrasion was used as the control (CON). All samples were primed prior to acrylic resin polymerization. After the resin was cured, the SBS was tested. The obtained data were analyzed by using multivariate analysis of variance(α = 0.05). After the SBS test, the interface failure modes were observed by scanning electron microscopy. The MS exhibited significantly higher bond strength after thermal cycles (*p* < 0.05) than the CON. Nevertheless, statistically comparisons resulted in no significant effect of the differently sized microslits on SBS (*p* > 0.05). Additionally, MS (before thermal cycles: 34.8 ± 3.6 to 35.7 ± 4.0 MPa; after thermal cycles: 26.9 ± 3.1 to 32.6 ± 3.3 MPa) demonstrated greater SBS and bonding durability than that of CON (before thermal cycles: 17.3 ± 4.7 to 17.9 ± 5.8 MPa; after thermal cycles: 1.0 ± 0.3 to 1.7 ± 1.1 MPa), confirming that the micromechanical retention with laser-milled microslits was effective at enhancing the bonding strength and durability of the acrylic resin and zirconia. Polycrystalline zirconia-based ceramics are a newly accessible material for improving removable prosthodontic treatment, as the bond strength with acrylic resin can be greatly enhanced by laser milling.

## 1. Introduction

Metal alloys, such as cobalt-chromium, titanium, and gold, are widely used in removable prosthodontic treatment as materials for the frameworks of removable particle dentures (RPDs). These metal alloys possess favorable characteristics, such as excellent mechanical strength and thermal conductivity. However, the demand for metal-free treatments has increased due to various factors, such as escalating precious metal prices [1], concerns regarding metal allergies [2], and increased emphasis on aesthetics [3]. Moreover, due to factors such as health, anatomy, psychology, and economics, many patients are not ideal candidates for implant therapy. Particularly in geriatric dentistry, invasive treatment may cause a physiological burden, which can potentially result in postoperative complications [4,5]. Therefore, RPD treatment may reduce the physical and mental burden on elderly patients, before and after treatment, and additionally rehabilitate the oral function and improve the psychological and social adaptation. 

Enhanced technology, such as computer-aided design and computer-aided manufacturing (CAD/CAM), has been successfully integrated into dentistry from the engineering sciences. The polycrystalline zirconia-based ceramics with superior mechanical properties, which can only be processed by CAD/CAM, have been widely utilized in clinical dentistry [6]. Zirconia ceramics are attracting attention as biomedical materials because of their favorable characteristics, including excellent biocompatibility, high chemical stability, and mechanical strength comparable to that of metals [6,7,8]. The typical zirconia ceramic used in dental prostheses is yttria-stabilized tetragonal zirconia polycrystal (Y-TZP) [9,10]. However, ceria-stabilized tetragonal zirconia/alumina nanocomposite (Ce-TZP/A) has recently attracted attention in prosthetic dentistry, due to its coequal flexural strength (600–800 MPa) with Y-TZP (>900 MPa) and higher fracture toughness (~19 MPa m1/2) than Y-TZP (5–10 MPa m1/2) [11]. Based on these superior material properties of zirconia ceramics, they are considered suitable candidates for replacing the metallic components of RPDs. Despite the fact that many scholars have confirmed that zirconia ceramics are a suitable alternative for the RPD framework [12,13,14,15], studies related to the adhesive properties of acrylic resin and zirconia ceramics are limited. 

Previous literature has reported that zirconia ceramics are potentially alternative materials for connectors, clasps, and other components used in the framework of RPDs [14,15] due to excellent bending properties [12] and fatigue resistance [13]. Moreover, zirconia-based frameworks are light in weight, aesthetically pleasing, and do not present complications, such as metal allergies [13]. However, favorable mechanical properties are not the only considerations when contemplating the applications of zirconia ceramics on RPD frameworks, since the adhesive property of zirconia ceramics regarding acrylic resin (denture-based resins) is also a critical factor. Failure between materials is mainly due to the biofilm accumulated on the dental materials [16], and firm bonding promotes the long-term use of dental treatments, such as RPDs, as it reduces the likelihood of fractures along the margin edges, and the odor and discoloration that may be caused by saliva intrusion into the margin gap. Primers with functional monomers such as 4-META (4-methacryloyloxyethy trimellitate anhydride) or MDP (10-methacryloyloxydecyl dihydrogen phosphate) [17,18], and tribochemical treatment [19] are proposed for chemical preprocessing. Air-abrasion (alumina blasting) treatment has been generally endorsed as the mechanical procedure for creating irregularities on the adhesive interface of the materials [20]. Thus, a combination of chemical and mechanical preprocessing was confirmed to improve the bonding between zirconia ceramics and other substrate materials [21,22]. However, previous work indicates that on account of the high-hardness of zirconia ceramics, the superficial irregularities formed by conventional pretreatment methods are limited [23]. 

In the last decade, with the advancement of laser techniques, the use of lasers as an alternative pretreatment method for dental procedures has become prevalent, such as by applying laser treatment to dental materials to improve the bonding properties between materials and resin-based luting agents [24] or materials and porcelain [25]. This resulted in the attempted use of lasers as a surface treatment method for zirconia ceramics, in order to enhance the bonding strength and durability of a number of dental materials by altering the surface microstructures [26,27,28]. Some studies have reported that lasers improve the bond strengths of zirconia ceramics and other substrate materials; however, other studies have shown contradictory results [29,30,31]. Furthermore, studies evaluating the bonding between zirconia ceramics and acrylic resin are limited; there is only one related published study on the effect of microslit retention on the bond strength of zirconia to dental materials [25]. Therefore, research in the area of laser-milled microslits is critical for improving RPDs and will ultimately serve the future of clinical dentistry.

Herein, we aim to investigate the effects of micromechanical retention (microslits of different sizes) created by lasers on durability and bond strength regarding two different zirconia ceramics and an acrylic resin. The theory is that (1) laser pretreatment should increase the shear bond strength (SBS) and durability of the zirconia ceramics and acrylic resin; (2) the different sizes of microslits (laser grooves) will alter the SBS; and (3) the SBS would be unchanged after artificial aging via thermal cycling.

## 2. Materials and Methods 

The details of the materials used in this study are provided in Table 1. The experiment was conducted using the following procedure:

### 2.1. Zirconia Specimen Preparation

**Figure 1:**
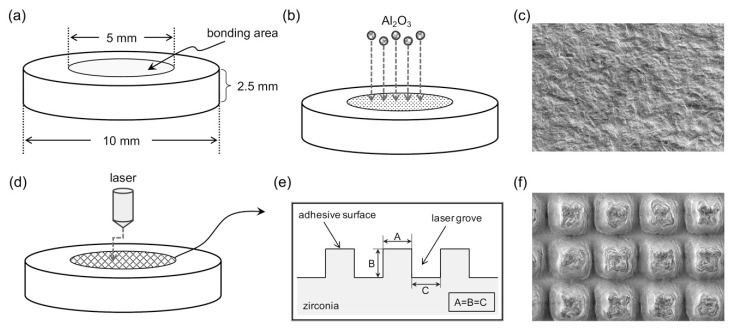
Schematic illustrations of the specimens. The design of the zirconia ceramic disk-shaped specimens with the bonding area at the middle of the specimens (**a**). Two different surface treatments of zirconia ceramic groups: (1) air-abrasion treatments (control (CON)) with 50 μm alumina particles (Al_2_O_3_) and 0.3 MPa jet pressure for 10 s with the distance from the orifice to the zirconia surface of 10 mm (**b**), and the scanning electron micrograph (SEM) of the specimen surface (**c**); (2) Nd: YVO_4_ laser treatments (**d**), an isometric grid pattern (A = B = C) with groove dimensions of 50, 75, and 100 μm (**e**), and SEM of the specimen surface (**f**).

Two types of zirconia ceramics (Y-TZP and Ce-TZP/A) were processed with the CAD/CAM system and then sintered in a high-temperature furnace. The temperature was raised to 1450 °C and maintained for 2 h. The zirconia ceramics were then fabricated into disk-shaped specimens with a diameter of 10 mm and a thickness of 2.5 mm (Figure 1a). 

### 2.2. Surface Treatment

Eighty disk-shaped specimens were prepared for each zirconia ceramic. All specimens were then ground flat with 600-grit silicon carbide abrasive paper, followed by steam cleaning and air-drying. Subsequently, specimens were randomly distributed into four groups according to the surface treatments (n = 20 per group for each zirconia ceramic).

Specimens of air-abrasion pretreatment (group control (CON)) were treated by air-abrasion with grain-sized alumina (Al_2_O_3_) particles of 50 μm, and jet pressures of 0.3 MPa, for 10 s. The distance from the orifice to the zirconia ceramic’s surface was approximately 10 mm (group CON), as shown in Figure 1b,c. The microslits of laser pretreatment (group MS (microslits)) were fabricated by using a neodymium-doped yttrium orthovanadate (Nd: YVO_4_) laser (LASERTEC 40; Mori Seiki Co., Ltd., Nagoya, Japan). The laser irradiation conditions were 70 kHz frequency, 1065 nm pulse-width, 60 mm irradiation distance, and 5–6 min radiation time. The microslits (laser grooves) followed an isometric grid pattern with groove dimensions of 50 μm (group 50MS), 75 μm (group 75MS), and 100 μm (group 100MS), as shown in Figure 1d to 1f.

### 2.3. Shear Bond Strength

A brass mold (6 mm inner diameter, 8 mm outer diameter, and 2 mm height) was positioned on the surface of each zirconia ceramic specimen to define the bonding area (Figure 1a) and was fixed by a piece of double-sided tape. Then, a ceramic primer was applied to the zirconia ceramic surfaces of all disks. Next, an acrylic resin was mixed in at the recommended liquid/powder ratio provided by the manufacturer and poured into a brass mold. Subsequently, the specimens were then polymerized at 55 °C and 0.2 MPa for 30 min using a pressure vessel (Palamat practice ELT; Kulzer Japan Co., Ltd., Tokyo, Japan). 

**Figure 2:**
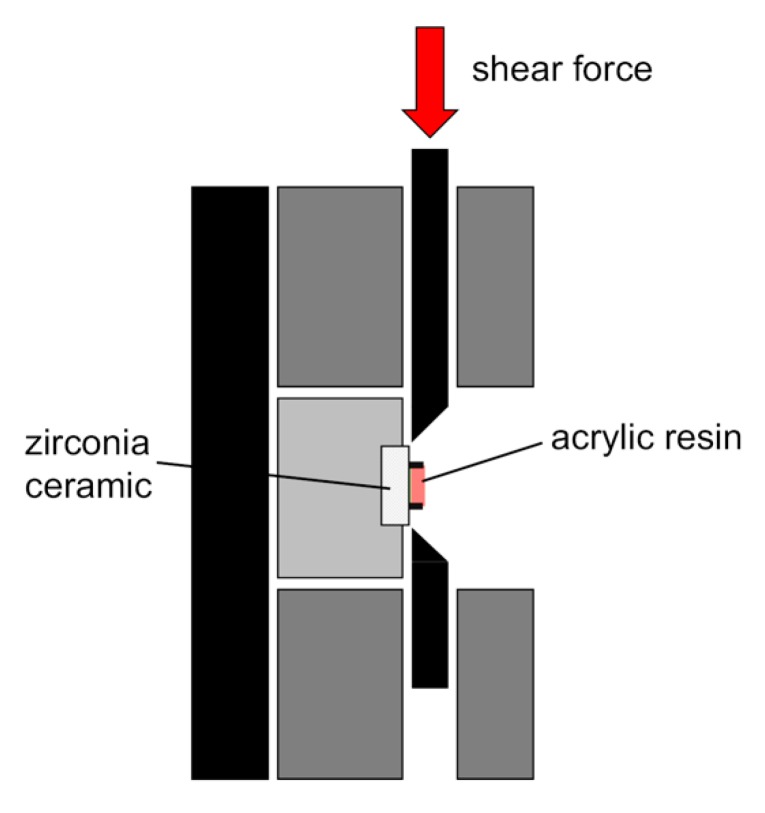
Schematic diagram of shear bond strength (SBS) testing and testing jig.

One hour after the bonding procedure, the specimens were immersed in water at 37 °C for 24 h. This state was defined as a “zero-thermal cycle.” The 24 h shear bond strength of half the number of specimens in each group (n = 10) was tested at the zero-thermal cycle. The remaining specimens (n = 10) were placed in a thermal cycling apparatus (Thermal Cycler; Nissin Seiki Co., Ltd., Tokyo, Japan) and cycled between a cold bath (4 °C) and a hot bath (60 °C) with a one-minute dwell time per bath for 20,000 cycles. The specimens immersed in water for 24 h (at the zero-thermal cycle) and those after 20,000 thermal cycles were subjected to SBS testing. The specimens were attached to a shearing jig (Figure 2) and the SBS test was conducted using the universal testing machine (Autograph AGS-J; Shimadzu Corp., Kyoto, Japan). A shear force was applied to the adhesive interface until fracture occurred at a crosshead speed of 0.5 mm/min. The load value of the debonded zirconia ceramics and the acrylic resin was then measured.

After the SBS test, the fractured interfaces were observed with an optical microscope (S300II; Inoue Attachment Co. Ltd., Tokyo, Japan) at 8× magnification to determine the mode of failure. The failure modes were classified into (1) adhesive failure (no residual resin on the zirconia ceramic surface, and it is cleanly delaminated at the zirconia–resin interface), (2) cohesive failure (zirconia ceramic and resin are firmly bonded; cracks inside the resin cause debonding), and (3) mixed failure (combination of cohesive and adhesive failure). The fractured surfaces were also observed by scanning electron microscopy (SEM) at 500× magnification (VE-8800; Keyence Corp., Osaka, Japan), at a high vacuum level and an operating voltage of 2 kV.

### 2.4. Statistic Analysis

The data collected for all tests were calculated, and the homogeneity of variance was primarily analyzed using Levene’s test. Data comparisons were conducted using one-way analysis of variance (ANOVA) with post hoc Scheffé test and post hoc Tukey’s HSD (honestly significant difference) tests. All statistical analyses were performed using IBM SPSS statistical software (SPSS version 24; IBM, Armonk, NY, USA), and the level of statistical significance was set at 5%.

## 3. Results

### 3.1. Shear Bond Strength (SBS) Testing

Table 2 illustrates the results of SBS testing of Y-TZP. Irrespective of artificial aging, CON had a significantly lower SBS than MS. Prior to artificial aging, the SBSs were 34.8 ± 3.6 to 35.7 ± 4.0 MPa for MS, and 17.9 ± 5.8 MPa for CON. After artificial aging, there was a 94.3% reduction in SBS values, with a significant decline from 17.9 ± 5.8 MPa to 1.0 ± 0.3 MPa for CON (*p* < 0.05). However, in MS there was no dramatic reduction (<25%) observed in the SBS values irrespective of the artificial aging process or the different conditions of laser treatment (50 MS, 75 MS, or 100 MS). 

The results of SBS testing of Ce-TZP/A follow a similar trend to those of Y-TZP, as seen in Table 3. The SBS of CON was significantly lower than that of MS, regardless of whether artificial aging was performed or not. Before artificial aging, the SBSs were 34.9 ± 4.3 to 35.2 ± 4.0 MPa for MS, and 17.3 ± 4.7 MPa for CON. Post aging, the SBS values of CON significantly decreased to 1.7 ± 1.1 MPa, corresponding to a reduction of 90.2% (*p* < 0.05). However, no dramatic reduction (< 9%) in SBS values occurred in MS regardless of the different conditions of laser treatment (50 MS, 75 MS, or 100 MS), or whether or not artificial aging was performed.

### 3.2. Mode of Failure

**Figure 3:**
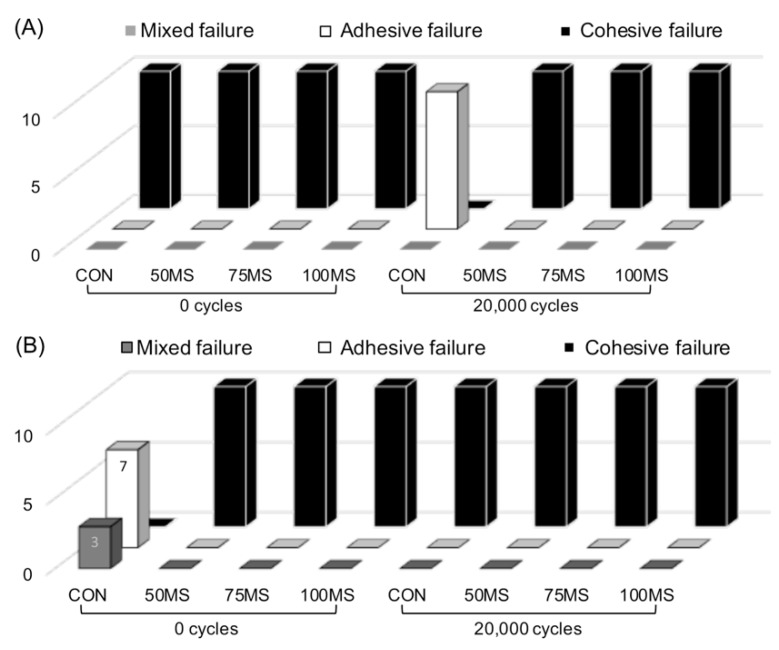
Failure mode of (**A**) Y-TZP, (**B**) Ce-TZP/A after shear bond strength (SBS) testing. Mixed failure: combination of cohesive and adhesive failure; 0 cycles: before artificial aging; 20,000 cycles: after artificial aging.

Figure 3 illustrates the classification of the failure mode post SBS testing. For both types of zirconia ceramic, MS displayed cohesive failure irrespective of whether or not artificial aging was performed. Conversely, adhesive failures were predominant in the controlled samples (CON).

**Figure 4:**
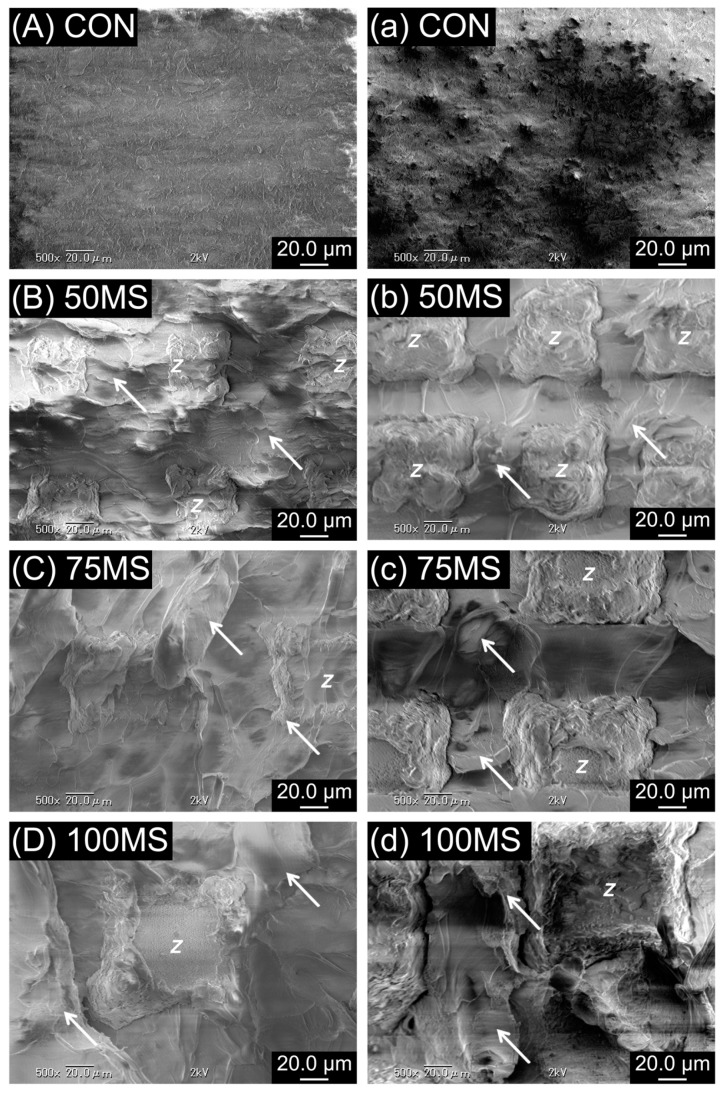
Scanning electron micrographs (SEM) of debonded Y-TZP and acrylic resin interface before (**A**–**D**) and after artificial aging (**a**–**d**). The arrow in the figure refers to the residual acrylic resins, while the Latin script letter Z refers to zirconia ceramics; original magnification 500×.

**Figure 5:**
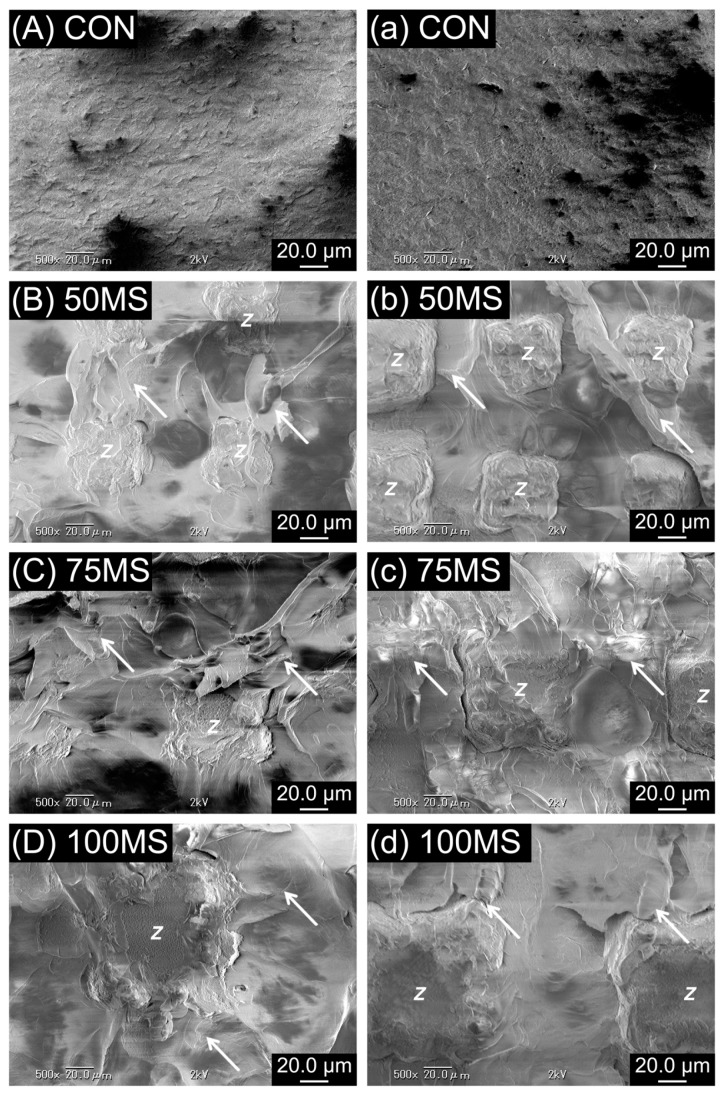
Scanning electron micrographs (SEM) of debonded Ce-TZP/A and acrylic resin interface before (**A**–**D**) and after artificial aging (**a**–**d**). The arrow in the figure refers to the residual acrylic resins, while the Latin script letter Z refers to zirconia ceramics; original magnification 500×.

From the representative SEM images of the debonded zirconia-resin interface (Figure 4 and Figure 5), it was observed that the resin penetrated the microslits of MS. As a result, analysis of the debonded interface visibly displayed resin residue on the zirconia ceramic surface. In contrast, no residual resin was observed for CON.

## 4. Discussion

The shear bond strengths of the acrylic resin and two types of zirconia ceramic (Y-TZP and Ce-TZP/A) were evaluated after traditional surface pretreatment, air-abrasion with 50 μm and 0.3 MPa Al_2_O_3_ particles (CON) [23], and a novel pretreatment, using a laser to create microslits (MS). Despite the fact that studies on the bonding strength between acrylic resin and metal alloys [32,33] postulate that bonding strength is sufficient for clinical dentistry, the durability after thermal cycling as a means of artificial aging, requires discussing. The results confirmed that the SBS values of MS were higher than that of CON, regardless of artificial aging. Consequently, this means that the first null hypothesis was accepted. A contrasting explanation is that the microslits created by the laser provide a higher mechanical interlocking force due to the more profound irregularities than air-abrasion. Yamaguchi et al. [34], reported that rougher surfaces have more extensive contact areas available for bonding. The current study did not conduct experiments pertaining to the surface roughness; however, other published papers have reported that the surface roughness of the air-abrasion with Al_2_O_3_ (under identical conditions) was approximately 0.23 μm for Y-TZP and 0.24 μm for Ce-TZP/A [23]. Therefore, it is understood that due to the uneven and rough surface, CON creates shallower surface defects than the microslits (50 MS, 75 MS or 100 MS). Hence, the contact area of CON was smaller than that of MS, resulting in a lower SBS in CON than in MS. The findings of this study confirmed that laser treatment is an effective mechanical process that can improve the bonding strength of acrylic resin and zirconia ceramics.

Compared with the traditional mechanical surface treatment methods, laser treatment is suggested to be more controllable and stable. Thus, lasers have been widely used in medicine over the past decade. In dentistry, the erbium-doped yttrium-aluminum-garnet (Er: YAG) laser is the most frequently used laser system. Based on prior related experiments, it was determined that although Er: YAG lasers can improve the surface roughness of zirconia ceramics [30], they result in decreased bond strength [31]. Usumez et al. [26], concluded that neodymium-doped yttrium-aluminum-garnet (Nd: YAG) lasers cause microcracks in zirconia ceramics and might reduce the resistance and longevity of the material. However, computer numerical control (CNC) lasers are widely used in the field of engineering science for tasks such as the manufacturing of molds or lettering, and the production of microscopic surface textures on various materials. Therefore, we utilized a CNC laser with neodymium-doped yttrium orthovanadate (Nd: YVO_4_) to make microslits on the surface of zirconia ceramics [25]. The reproducibility of the microslit topography was improved by adjusting laser parameters [25]. Three isometric grid microslits (Figure 1) of different sizes (50, 75, and 100 μm) were created on the surface of the zirconia ceramics in order to analyze the effect of microslit size on bonding strength. The experimental results show that although the sizes of the microslits influence the contact area, with the 50MS exhibiting the largest contact area of bonding (when 100MS set to 1, 75MS would be 1.21, and 50MS would be 1.27), differences in size did not affect the SBS values. Therefore, the second null hypothesis is rejected. The proposed reason for the observed trend is that although the surface areas of the three microslits vary slightly, the influence of microslit size on the bonding strength is imperceptible due to similarity in microslit topography. Previous research on the effect of different conditions of air-abrasion to SBS also indicated similar results; although increasing the particle size or the jet pressure of the air-abrasion caused a significant increase in surface roughness, there was no apparent effect on SBS and bonding durability [23]. Therefore, it is possible to conclude that the bond strength would potentially remain unchanged, despite increasing the size of the microslit.

Previous studies have reported that when acrylic resin bonds to other materials, the insufficient bonding strength at the adhesive surface causes microbreakage due to differences in the thermal expansion coefficient between the two substrates [35]. During artificial aging, the physical properties of the acrylic resin itself decreased due to water absorption. Moreover, due to massive differences in the thermal expansion coefficient between the acrylic resin and zirconia ceramics, there is induced water penetration in the adhesive layer and the stress at the interface is increased [36]. Furthermore, the bond durability declines, and debonding occurs. The SBS values of CON after artificial aging had a significant decrease (> 90%) for Y-TZP and Ce-TZP/A, while SBS values of MS were not dramatically different for either substrate. The results proved the third null hypothesis, which states that the SBS values remain unchanged after artificial aging. One possible explanation is that when the acrylic resin became polymerized under pressure, the molecules were polymerizing towards the surface of the zirconia ceramics and were deeply interlocked into the microslits created by the laser (MS). Thus, even after artificial aging, the acrylic resin absorbed water and nonhomogeneous thermal expansion occurred between the two materials. However, because of the physical fitting, the material successfully overcomes the peeling and debonding, thereby improving the bonding durability. All specimens in MS showed cohesive failure, which suggests that the bonding strength was more significant than the strength of the acrylic resin structure, and thus fracture and failure occurred within the acrylic resin. Most CON specimens showed adhesive failure due to insufficient bond strength. The reason for failure can be attributed to insufficient resin penetrating the undercut caused by the air-abrasion treatment. When shear force was applied, debonding directly occurred at the zirconia–resin interface, which can be confirmed by the SEM images.

The minimum acceptable SBS value is 5 MPa at the interface of resin-based materials and substrate, as specified in ISO 10477 [37]. Additionally, others have suggested that the bonding strength should be greater than 5.9 to 10.0 MPa to adequately satisfy routine clinical use [38,39]. When acrylic resin bonds to a metal alloy (after priming and air-abrasion), the literature SBS values obtained after thermal cycling were 2.48 ± 0.66 MPa (cobalt-chromium alloy), 1.96 ± 0.78 MPa (titanium alloy), and 1.07 ± 0.74 MPa (gold alloy) [17]. In the case of acrylic resin bonded to zirconia ceramics, as shown by Iwaguro et al. [25], even after priming and air-abrasion treatment, the SBS values obtained after thermocycling could not be evaluated (0 MPa). The results of the present study are in accordance with the previously mentioned reports (SBS values < 2 MPa) and infer that the bond strength might not be sufficient for long-term usage. Nevertheless, when the surface of zirconia ceramic contains microslits (MS), all the SBS values of both Y-TZP and Ce-TZP/A before (> 35.1 ± 2.1 MPa) and after (> 26.9 ± 3.1 MPa) artificial aging exceed the required SBS values (5–10 MPa) recommended in the literature. Laser pretreatment has more than doubled the bond strength of acrylic resin to zirconia ceramics, and preventing failure after artificial aging. Therefore, laser pretreatment is proposed as an alternative to mechanical processing because of its ability to effectively improve the bonding durability and strength between denture acrylic resin and zirconia ceramics in clinical dentistry. 

Additional research is required to determine the thickness of the RPD framework made by zirconia ceramics and combine it with microslits. Furthermore, the processing time and the strength of the Nd: YVO_4_ laser also needs to be assessed in future studies. CNC lasers are not popularly used in dentistry due to their size, so a reduction in size, such as a desktop CNC laser machine, is also an essential issue that requires further investigation. Furthermore, when applying laser as a surface treatment method, the high temperature and heat of laser might cause microstructure change in zirconia ceramics and might further affect the material properties; thus, the internal structure changing mechanism of materials should also implement in future studies. Regardless, the present study provides substantial evidence to support the application of Nd: YVO_4_ lasers in dentistry, in order to substantially improve the adhesive properties of acrylic resin bond to zirconia ceramics.

## 5. Conclusions

The limited results of this in vitro study prompt that micromechanical microslits etched with a Nd: YVO_4_ laser effectively improve the bonding strength and durability of acrylic resin bond to zirconia ceramics. After artificial aging, the bond strength of the air-abrasion treated zirconia ceramics (CON) decreased, but that of the laser etched materials (MS) remained high. Yet, there was no difference in the bond strength between the different sizes of microslits diameter (50, 75, and 100 μm).

## Figures and Tables

**Table 1:** List and composition of the materials used.

Material/Identification (Abbr.)	Manufacturer	Main Composition *	Lot No.
Zirconia ceramic			
TZ-3YB-E (Y-TZP)	Tosoh Corporation, Tokyo, Japan	ZrO_2_, Y_2_O_3_	S305374B
Cpro NANOZirconia (Ce-TZP/A)	YAMAKIN Co., Ltd., Osaka, Japan	ZrO_2_, Al_2_O_3_, CeO_2_	CD90011076J
Acrylic resin			
PalaXpress^®^ Ultra	Kulzer Japan Co., Ltd., Tokyo, Japan	PMMA (Powder)	012030
		MMA (Liquid)	010233
Primer			
Clearfil^®^ Ceramic Primer Plus	Kuraray Medical Inc., Tokyo, Japan	MDP, silane, ethanol	7R0018
Alumina particle			
Cobra	Renfert GmbH, Hilzingen, Germany	Al_2_O_3_, SiO_2_	700388

PMMA, polymethyl methacrylate; MMA, methyl methacrylate; MDP, 10-methacryloyloxydecyl dihydrogen phosphate. * According to information provided by manufacturers.

**Table 2:** Mean shear bond strengths (MPa) of acrylic resin bonded to Y-TZP.

Conditions	0 Thermal Cycles	20,000 Thermal Cycles	Reduction
	Mean ±SD	Mean ±SD	
CON	17.9 ± 5.8 ^a^	1.0 ± 0.3 ^A^	94.3%
50 MS	35.7 ± 4.0 ^b^	26.9 ± 3.1 ^B^	24.8%
75 MS	34.8 ± 3.6 ^b^	28.7 ± 4.2 ^B^	17.8%
100 MS	35.1 ± 3.5 ^b^	27.6 ± 4.5 ^B^	21.1%

SD, standard deviation; within same column, different letters indicate groups that are statistically different (*p* < 0.05); Reduction, rate of reduction.

**Table 3:** Mean shear bond strengths (MPa) of acrylic resin bonded to Ce-TZP/A.

Conditions	0 Thermal Cycles	20,000 Thermal Cycles	Reduction
	Mean ±SD	Mean ±SD	
CON	17.3 ± 4.7 ^a^	1.7 ± 1.1 ^A^	90.2%
50 MS	35.1 ± 2.1 ^b^	32.0 ± 3.5 ^B^	8.9%
75 MS	34.9 ± 4.3 ^b^	32.2 ± 4.1 ^B^	7.7%
100 MS	35.2 ± 4.0 ^b^	32.6 ± 3.3 ^B^	7.3%

SD, standard deviation; within same column, different letters indicate groups that are statistically different (*p* < 0.05); Reduction, rate of reduction.
